# Memory recall: New behavioral protocols for examining distinct forms of context specific recall in animals

**DOI:** 10.1016/j.nlm.2022.107685

**Published:** 2022-09-26

**Authors:** A. Prodan, H. Davies, H. Eneqvist, G. Mastroberardino, H. Wijayathunga, K. Wardlaw, R.G.M. Morris

**Affiliations:** Laboratory for Cognitive Neuroscience, Centre for Discovery Brain Sciences, Edinburgh Neuroscience, University of Edinburgh, 1 George Square, Edinburgh, EH8 9JZ, United Kingdom

**Keywords:** context specific recall, episodic memory, event arena, allocentric spatial representation, context-specific recency

## Abstract

This study outlines two novel protocols for examining context specific recall in animals prior to embarking on neurobiological studies. The approach is distinct from and contrasts with studies investigating associative familiarity that depend upon procedural variations of the widely used novel object recognition task. It uses an event arena in which animals are trained across numerous sessions to search for, find and dig up reward from sandwells during sample and choice trials – a prominent spatial event for a rodent. The arena could be laid out as either of two highly distinct contexts with which the animals became fully familiar throughout training. In one protocol, the location of the correct sandwell in each context remained stable across days, whereas in the other, the correct digging location varied in a counterbalanced manner across each successive session. Thus, context-specific recall of the spatial location of successful digging during choice trials was either from a stable long-term memory or could reflect context specific spatial recency of the location where reward had been available that session. Both protocols revealed effective memory recall in choice and probe tests which, at the point of test, were procedurally identical in both cases.

## Introduction

The concept of ‘context-specificity’ in memory is discussed extensively in experimental psychology and neuroscience (Nadel & Maurer, 2020). It can carry different meanings, ranging from remembering where objects are located within a context or where events happen, through to non-spatial facets such as social context. When context is spoken about colloquially, it is generally in the domain of recall rather than recognition, as in “*Where do I go to find the Empire State building?*” or “*When or where did something happen?*”. The answer is likely to involve recall, with a reply to the former involving recall from a stable long-term spatial memory but a reply to the latter having a more episodic character.

Context has been examined experimentally in neurobiological animal studies of memory for many years. Prominent examples include its role in access to reward (or punishment). Cues are present associated with recognizing a context or another, and the task is to associate a biologically significant event (e.g. shock) with one or another recognised context. This is the simplest form of context discrimination of which the paradigmatic example is context fear conditioning (Anagnostaras *et al*., 1999; Kim *et al*., 1991). This protocol is now used in numerous ‘gain-of-function’ optogenetic studies pointing to a role for the dentate gyrus in contextual identification (Josselyn & Tonegawa, 2020). In other studies, a context sets the occasion for some other stimulus to be rewarded but that same stimulus goes unrewarded in a different context, and vice versa — a context-specific stimulus discrimination task in which one stimulus was repeatedly rewarded in one context and another non-rewarded, and these reward assignments reversed in the other context (Good & Honey, 1991). Learning such a task has been shown to depend on the integrity of the hippocampus. Context may also define the presence of objects that animals can recognize and investigate, such as discriminable familiar and novel objects during an initial exploratory sample trial in a context to which the animals have previously been habituated (Ennaceur & Aggleton, 1994; Ennaceur & de Souza Silva, 2018). In a subsequent choice trial, one (or more) of these objects are either temporarily absent, replaced by another novel object, moved to a different context, or moved to a different location in the same or a different context (Dix & Aggleton, 1999). Such procedural manipulations alter the ‘associative’ familiarity or novelty of these objects relative to the sample trial. Some detailed novel object protocols have examined ‘context-specific’ novelty (Langston & Wood, 2010) and its relevance to, for example, neurodevelopmental disorders (Asiminas *et al*., 2019), but have tended to do so over relatively short memory intervals (e.g., 3 min for Langston and Wood, 2010). Examining the contribution of context to recognition memory is central to a wide range of work, the typical result being that the experimental animal engages in renewed exploratory behavior in the incongruous setting relative to the congruent one, but at the expense of using only relatively short memory retention intervals. Spontaneous exploration has, nonetheless, revealed much about the anatomical organisation of recognition memory (Aggleton & Nelson, 2020; Aggleton & Pearce, 2001).

In contrast, our interest in the present work centers initially on the ability to recall rather than merely recognise in a context-specific manner. Recall about where something is located or what happened in a specific context is not only frequent in everyday life, but also more common than recognition in ordinary discourse; a casual question from someone about whether one can remember something is rarely accompanied by a forced choice test of alternatives, except perhaps in a police line-up. A second key idea is that recall from a stable long-term memory of facts (‘semantic-like’ recall) is likely different from remembering something that happened recently (‘episodic-like’ recall). For example, you likely recall where and when you last saw someone. Episodic recall engages a process of something ‘coming to mind’ from the past, but it is important to realize that recall may not always be ‘episodic’. It may also be from a stable long-term memory – more akin to recall from semantic memory.

This important distinction pertains to animal tasks as well. Navigation in the standard watermaze task involves recall but it is not episodic and does not require mental time travel (Morris *et al*., 1982). The hidden escape platform is generally in a fixed location across trials and the animal’s swim trials start at each of the four cardinal locations around the pool (N, S, E and W) in quasi-random sequence. After approximately 10 days of training, the animal can be placed in the pool at any location, and it will navigate using a relatively direct path to the escape location. There is no local cue associated with the hidden escape platform – in effect nothing to ‘recognise’ beyond the context which itself provides no explicit guidance cues about where to go. The watermaze limits the use of path-integration by animals (because there is no return path) and, with this type of standard training from multiple start locations, the spatial representation is definitively allocentric. Note that there is no need for the animal at the start of a trial to remember what it did in the pool on some prior occasion. With respect to context specificity, watermaze navigation to a stable planned destination may also be achieved by a learned and relatively direct route that differs from one context to another (Bannerman *et al*., 1995). In these training protocols, it is *not* an ‘episodic-like’ memory task.

Other protocols in the watermaze are, however, more ‘event-like’ as outlined and discussed by Steele and Morris (1999). The key change in that study was to move the correct location of the hidden platform each day such that the animal could not know the correct location of the first daily trial. However, the same study showed that the new daily location could be learned in one trial. This study was later followed by similar protocols in the event arena (Bast *et al*., 2005). Memory recall has, however, been more exactingly investigated in ‘episodic-like’ memory tasks such as ‘what-where-when’ food choice after relatively long memory delays in corvids (Clayton & Dickinson, 1998) and rats using carefully designed radial-maze tasks (Babb & Crystal, 2006; Crystal, 2021). There are also tasks testing whether absolute time of memory encoding or the passage of time is recalled at the point of retrieval with data indicating that either may be used depending on procedure (Roberts *et al*., 2008; Zhou & Crystal, 2009). These tasks may, however, not be the only way of thinking about temporal ‘recency’. Another concept, first introduced in the human literature by Morton and colleagues with reference to the concept of “headed records”, explores the memory representations of events for the last meeting between two individuals – whether be it recently or much longer ago (Morton *et al*., 1985). It is this sense of ‘recency’ that is explored in this study.

**Figure 1:**
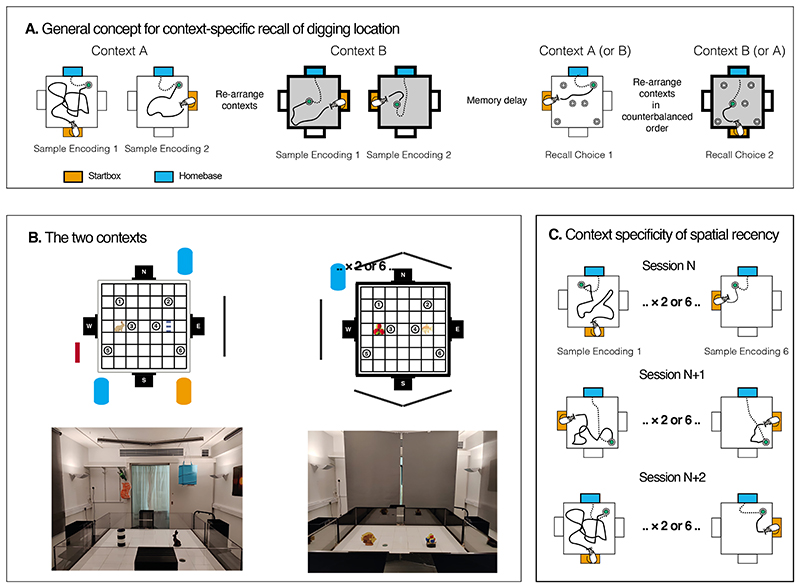
The apparatus and protocol concept. **A)** A set of 2 sample trials are scheduled each for each of two distinctive contexts followed by 2 choice trials, one in each context (and conducted in a counterbalanced order). As shown, the rewarded sample sandwell for context A (white) is in the north-east corner, and that for context B (grey) is in a south-east location. The startbox for each trial is shaded orange and the animal carries the food it has dug up to the home-base goal-box (blue). During each sample and choice trial, the animal retrieves one 0.5 g pellet from the rewarded sandwell (continuous line), which is of a size that the animal spontaneously chooses to carry back to the safety of the home-base (blue) where it is consumed. The rat is then given the opportunity to retrieve a second pellet, which is also brought back to the home-base (dotted line). This repetition serves only to confirm the encoding of where the food is to be found in each context. **B)** Photos and layout of the two contexts. Note distinct extra- and intra-maze cues whose identity and location remains stable for all 49 sessions. **C)** The second Phase of training involved a similar training protocol of sample trials followed by a choice trial, with the key difference being that the location of the rewarded sandwell (sample and choice) changed across successive sessions (N, N+1, etc.). For the second Phase of training, each session comprised of either 2 or 6 sample trials per context, each trial allowing the animal to retrieve one pellet from the rewarded sandwell, followed by the confirmatory run from the home-base to aid encoding (as described for **A**).

We here outline two novel ‘recall’ protocols, as a prelude to later neurobiological studies with lesions or drugs, to examine these distinct facets of context-specific recall. Specifically, we look in Phase 1 at the learning and then recall of a stable, allocentrically-defined context-specific target location, unmarked by local cues, to which an animal must go to secure reward (non-episodic); and in Phase 2 at the recall of the most recent location of a target whose position in a test arena changes daily in an apparently unstable manner (episodic-like). Although previously developed paradigms involving the use of classical and instrumental conditioning, or spatial learning protocols such as the watermaze, radial arm maze, or T-maze have been used to address certain facets of associative learning and spatial navigation, their utility in investigating episodic-like memory is limited because there is no demonstration of either information in context, nor that this information is changing over time. This motivated the development of our novel behavioral protocols using the event arena, a specifically designed customizable platform that permits disentangling event encoding and recall as well as long-term (multiple days) and short-term (several hours) context-specific object-location associations. The animals are trained in these sample trials to approach a cryptic sandwell containing hidden reward from which they can retrieve and then carry food to an allocentrically defined homebase to eat (Fig. 1A; Broadbent *et al*., 2020). The sample trials occur in rapid succession but do not involve discrimination between correct and incorrect locations; memory of where digging is occurring on each trial may nonetheless be incidentally encoded. The sample trials are followed, circa 1.5 h later, by a ‘choice trial’ in each context in which there are 6 sandwells with only the correct sandwell in that context being rewarded (6-alternative forced-choice, win-stay strategy; Fig. 1B). The choice trial provides a measure of memory recall. In a two-phase study, animals began Phase 1 by performing 2 sample trials (from either S, E or W) to a *stable* location in each context for 20 training sessions and 3 probe tests. The animals then performed one choice trial in each context per session, with the rewarded location in context A and that in context B being different but stable across sessions. An additional purpose of this phase was to provide clear evidence that animals could distinguish the contexts, a point often assumed but not directly tested in other studies. In the second Phase of the study, also consisting of 20 training sessions, we introduced the ‘recency’ dimension into the protocol. After demonstrating successful context-specific recall in Phase 1, the animals now had to remember in each session which was the most recently rewarded sandwell location in context A or context B. This location was randomly changed across sessions (Fig. 1C). By virtue of the animals now needing to recall where the correct sandwell was last found in each context, we conducted only one memory recall choice trial at the end of each day – and at a time-interval well beyond the domain of short-term memory. Whereas the former protocol is non-episodic, the latter is ‘episodic-like’ in Clayton and Dickinson’s (1998) terminology. A distinctive feature of the choice trials is that they are procedurally identical in both protocols; what differs is only the type of memory recall required of the animals.

## Results

**Figure 2:**
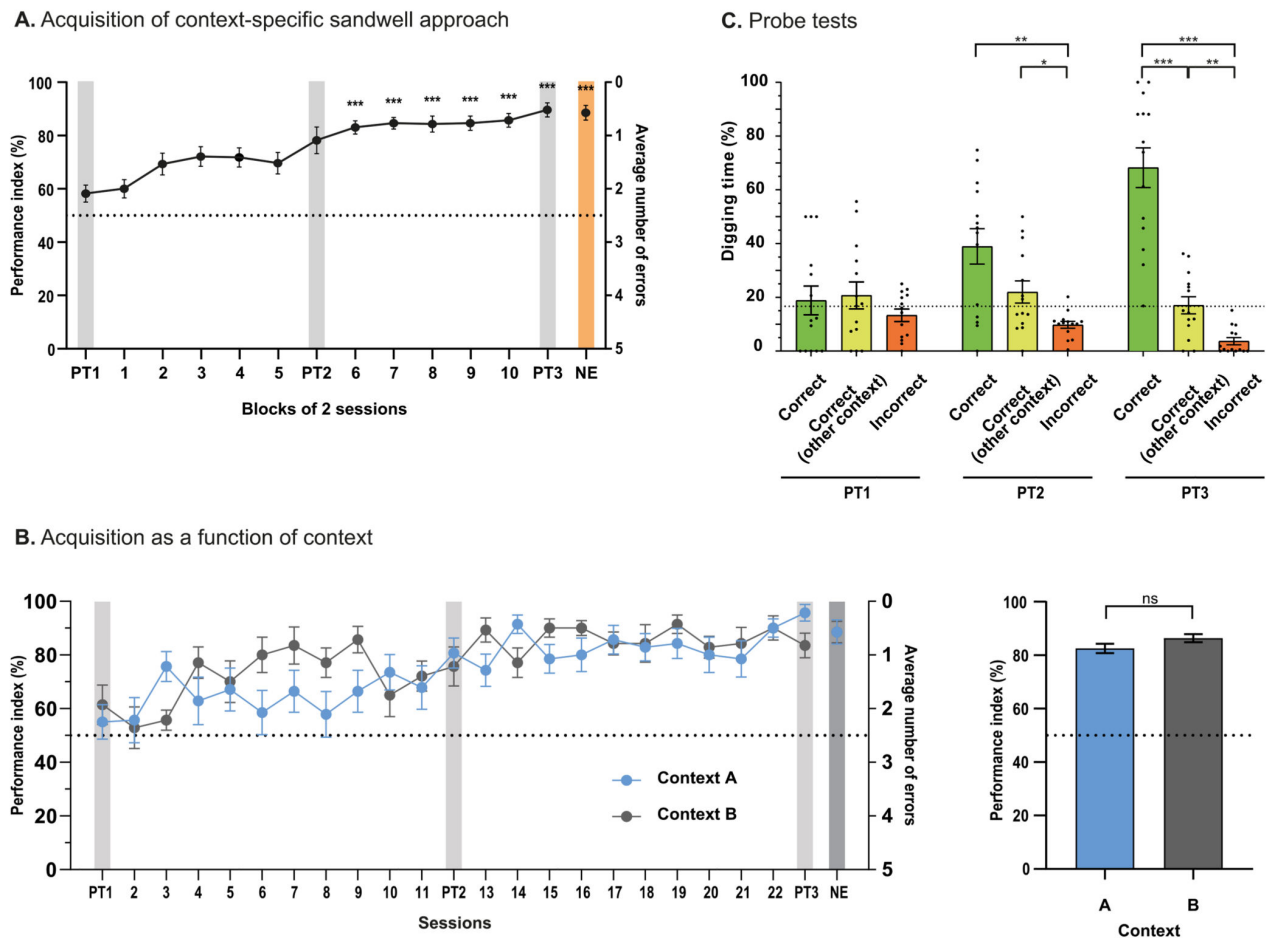
Context specificity with a stable sandwell location across sessions. **A)** Acquisition of the performance index for the choice trials for which 50% is chance performance and 100% represents no errors. A significant difference to chance developed early (by session 2) but the level of significance plotted only for the second half of training. Note steady gradual improvement of context-specificity. The non-encoding (NE) control session (no sample trials) is plotted with orange shading. **B)** There may have been a slight trend for context B to have been learned slightly faster than context A, but none of the quantitative statistics comparing the two contexts were significant. The significance level of performance during the last 10 sessions is shown for contexts A and B. Both are highly significantly above chance. **C)** Preferential digging at the contextually-specific correct sandwell rose from chance (PT1) to highly significant values in PT3. At no point was digging at the sandwell that would be correct in the other context ever above chance. Digging at the sandwells that were never rewarded gradually declined across sessions. * p < 0.05, ** p < 0.01, *** p < 0.001. Mean ± SEM.

The animals (N = 14; cohort 1 n = 8; cohort 2 n = 6) gradually learned the ‘stable’ Phase 1 recall protocol, beginning from a near chance level of choice trial performance (block 1) and rising to >80% correct over 10 blocks of training (blocks 7–10; 2 sessions/block; Fig. 2A). Individual animals learned to discriminate the two contexts and selectively approach and dig at the rewarded location in context A and the one in context B. A repeated measures ANOVA showed the increase in performance across sessions was highly significant (F(7.2, 194.3) = 12.48, p < 0.001, Greenhouse-Geisser correction). Furthermore, by blocks 6–10, the performance was highly significantly above chance (t(27) = 13.77, p < 0.001) with, on block 10, excellent performance and low variability (mean ± SEM; 85.71 ± 2.59%). This would be good performance in a 2-alternative forced-choice task and is exceptional for a 6-alternative task, indicating the accumulation of spatial knowledge across sessions (z score = 13.78). There may have been a trend to learn context B slightly earlier than context A but, overall, there was no significant interaction between context and blocks following asymptotic learning (S13–23; F(4, 108) = 0.734, p > 0.05; Fig. 2B). The final performance index score for contexts A and B over sessions 13–22 did not differ (Fig. 2B left; t(13) = 1.84, p > 0.05). Three separate probe tests (food reward absent, time spent digging measured over 60 s) were conducted at the start, mid-point, and end of training (sessions 1, 12, and 23, respectively). The probe tests were scheduled 1 h after the sample trials. The figure shows the striking growth of a significant interaction between probe trial and digging location (F(2.47, 32.23) = 9.38, p < 0.001) with a steady increase in accurate recall of the context-specific location discrimination. By PT3, the overwhelming proportion of time was spent digging at the contextually-specific correct location (68.23 ± 7.37% relative to chance at 16.67%; correct vs. chance, t(13) = 6.99, p < 0.001). There was a significant difference between digging time (%) in the correct sandwell and the incorrect sandwell(s) in PT2 (mean difference ± SE = 29.17 ± 7.65; 95% CI = 8.17– 50.16%, p < 0.01, Bonferroni; Fig. 2C) and PT3 (mean difference ± SE = 64.54 ± 8.62, 95% CI = 40.87–88.22%, p < 0.001, Bonferroni). While digging errors could occur to any of the other 5 sandwells, these errors could be categorically distinguished with respect to whether an error was to a sandwell location that was always incorrect or one that was correct in the other context, and the figure captures that distinction. In PT3, there was a significant difference between digging time (%) in the correct sandwell compared to that in the sandwell rewarded in the other context (mean difference ± SE = 51.21 ± 10.03, 95% CI = 23.66–78.75%, p < 0.001, Bonferroni). Moreover, when the data was examined with respect to performance in the opposite context, the sandwell correct in the other context was never above chance. There were, however, significant differences in time spent digging at the correct location in the other context and other incorrect locations (PT2: mean difference ± SE = 12.18 ± 4.34, p < 0.05; PT3: mean difference ± SE = 13.34 ± 2.85, p < 0.01). Observation of the digging time at the incorrect locations in the stable location Phase 1 probe trials reveals that the animals spent successively less time digging at locations that were never rewarded. Only two spatial locations were ever rewarded per animal, but the key point is that the choice of the correct location in the other context is never above chance, whereas the choice of the correct location in the appropriate context improved across probe tests reflecting the binding of spatial location to context in this task.

In the last session of Phase 1, a control procedure was conducted in which choice performance in the regular daily training protocol was tested in a single ‘non-encoding’ (NE) session. This involved not scheduling the normal sample trials prior to the choice trials (Fig. 2A). Despite the absence of sample trials, the animals’ choice performance in this NE control was excellent (mean = 88.57 ± 2.75%) and indistinguishable from that of block 10 and PT3 (Fig. 2A). This indicates that the animals had developed a long-term context-specific location memory that they could recall without needing the ‘reminder’ of the daily sample trials.

**Figure 3:**
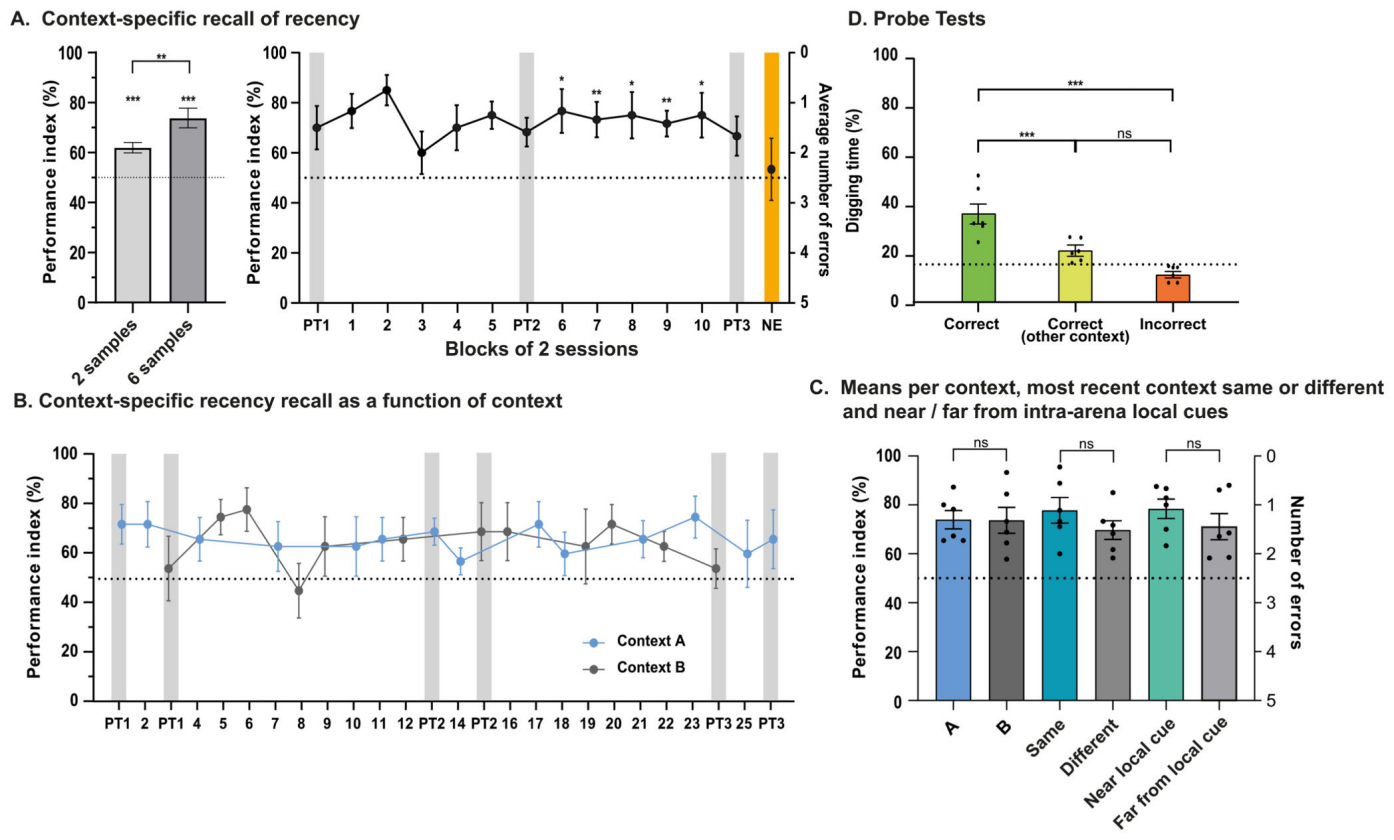
Context specificity of spatial recency recall across sessions. **A)** The now task-experienced animals were, on average, above chance in choosing the contextually-specific recent location of the sandwell as measured using the performance index for both the 2- and 6-sample protocol. The full data set is plotted across sessions for the 6-sample protocol. As in Phase 1, levels of significance are plotted for the second half of Phase 2 training. The non-encoding (NE) control session (no sample trials) is plotted with orange shading. **B)** No difference was observed in performance between contexts A and B. **C)** The full data was averaged across all sessions showing a performance index of circa 70% with minimal variability in both contexts (left). When performance in choice trials was plotted as a function of whether the animals were tested in the most recent sample-trials context or the least recent, no significant difference was observed (middle). When these same data were plotted as a function of sandwell proximity to the intra-arena cues, no significant difference was observed for near vs remote. **D)** In the probe trials, the percent time digging in the contextually-specific location of the earlier sample trials was well above chance. Again, percentage time digging in the correct location of the other context was at chance. * p < 0.05, ** p < 0.01, *** p < 0.001. Mean ± SEM.

We then turned to Phase 2, the episodic-like ‘recency’ protocol. With two exceptions, the task performed by the animals in this second Phase was identical to that of the earlier stable Phase (sample trials followed by choice). First, while we began by also having 2 sample trials, we later increased this to 6. Our findings indicated above-chance spatial recency memory with 2 sample trials (61.99% ± 2.08%; t(7) = -5.77, p < 0.001; Fig. 3A) and better absolute performance with 6 sample trials (73.83 ± 3.98% (t(5) = 5.99, p < 0.001; Fig. 3A). This improvement was significant (t(5) = 3.90, p < 0.01). The second exception was that on any one day/session, the choice trial was only tested in *one* of the two contexts but not *both* as in Phase 1 (the reason for this latter change being because, when choice trials were performed in both contexts in Cohort 1, performance in the choice trial in the second-tested context was influenced by the performance shown by the animals in the first context – likely a non-specific absolute recency effect. As we only wanted to measure context-specific recall of the most recent context-sample location association, choice trials were thereafter performed for only one context, with context A and context B being chosen in a random but counterbalanced order across sessions.

A consideration is: what do these data really represent? We considered three alternative explanations other than context-specific spatial recency. First, while we expected the scores reached at asymptote to be lower than that reached during the stable protocol (85%), both scores (62% and 74%) were good, above chance, and had lower inter-animal variability. However, performance might have been good in one context but not the other. The choice trial data for the 6-sample condition are therefore plotted as a function of the context in which recall was being expressed, revealing no difference between contexts A and B (t(5) = 0.07, p > 0.05; Fig. 3B and 3C-left). A second possibility was that animals were only displaying ‘absolute’ recency over time (Roberts *et al*., 2008), rather than ‘context-specific’ recency. A typical sequence of daily trials for an individual animal could have been sample trials (context A), sample trials (context B), choice trial (context A); or sample trials (context A), sample trials (context B), choice trial (context B). With the order of the contexts also counterbalanced across the sample trials, performance in the choice trial might have been dominated by high scores *only* when the context for the choice trial was the same as the *immediately preceding* context of the second set of sample trials, i.e. absolute recency, with poorer performance in recall of the earlier different context, that collectively average to above chance. In practice, we observed no difference in average performance between sessions in which the choice trial tested was in the ‘same’ context as the one used for the immediately preceding sample trials, i.e. ~15 min earlier, or in those given in the ‘different’ context of approximately 1.5 h earlier (Fig. 3C-middle; t(71) = -1.11, p > 0.05). This indicates that performance was guided by the recall of a context-specific recency memory rather than absolute recency. Third, we also considered whether alterations in the relative familiarity of the two intra-arena cues might contribute to apparent recall, guiding the animal to the vicinity of the correct sandwell. This seems unlikely as the animals were now familiar with all four intra-arena cues (two in each context) over as many as 23–43 sessions. Still, the prediction from a familiarity perspective might be that performance would be better for locations 3 and 4 in the arena (Fig. 1B), where the correct sandwell was directly beside an intra-arena cue, than for the other four more remote locations. We observed no such effect (Fig. 3C-right; t(5) = 1.17, p = 0.29).

Performance during the probe tests offered a further way to examine the context-specificity of recall. The measure is percent time digging at (a) the context-specifically correct sandwell, (b) the sandwell correct in the other context, or (c) the other sandwells that were incorrect on that session (but may be correct on other sessions). No accessible food was available. There was a significant difference between digging time (%) in the sandwells as a function of context-specific recency across all probe trials (6 sample condition: (F(2, 10) = 16.72, p < 0.001; Fig. 3D). Further, the digging time (%) in the correct sandwell was significantly different from that in the correct sandwell for the other context (mean difference ± SE; 14.63 ± 3.93, p < 0.001), and that in the incorrect sandwells for that session (mean difference ± SE; 24.23 ± 5.21, p < 0.001). However, as shown in Fig. 3D, no difference in digging time was observed between that in the correct sandwell for the other context and that in the incorrect sandwells (mean difference ± SE; 9.60 ± 3.29, p > 0.05). In this protocol, the incorrect sandwells are, of course, sometimes rewarded and thus the pattern of declining digging time in “incorrect” sandwells across training shown in Phase 1 does not occur.

Finally, as an internal control and unexpected test, we also conducted an KE session. Whereas in Phase 1, the prediction was that the daily sample trial would be *unnecessary*, the *opposite* prediction prevails for the recency Phase 2 as the correct daily location is defined by what happens in terms of memory encoding on the sample trials. As predicted, choice performance fell to chance (one sample t-test, NE vs chance; p > 0.05; Fig. 3A, orange shading, labelled NE). Moreover, an independent-samples t-test indicated a significant difference between the performance in the NE session in Phase 1 (mean ± SEM; 88.57 ± 2.75%) and that in Phase 2 (56.67 ± 14.06%; t(18) = -3.25, p < 0.01).

## Discussion

These novel protocols show that rats can readily (1) learn and later recall a *stable* context-specific spatial location of reward revealed as preferential approach to a different sandwell in each of two contexts; and separately (2) recall successfully the most *recent location* to approach in each context when the correct digging location is varied from day to day. In the latter case, access to a stable long-term memory provides allocentrically encoded information about the nature of each context, but the animal’s memory recall on the choice trial was of the most recent event of digging successfully for food in the contextually appropriate location.

One interest in developing these protocols was in exploring the transition from novelty-associated ‘recognition’ memory tasks to new ones based on ‘recall’. Recognition memory has been imaginatively modelled with ever more complex protocols that capture different facets of recognition – the absolute familiarity of an object, the relative familiarity/novelty of an object’s location, the formation of object-location associations and recognition of when these are misplaced in the same or different contexts (Aggleton & Nelson, 2020). In these tasks, the animal does not express either ‘correct’ or ‘incorrect’ behavior – it investigates an object and the extent to which it does so is measured quantitatively. The differential investigation of objects is then taken as a measure of the different forms of recognition memory dissociated largely through lesion studies complemented by studies measuring immediate early-gene activation (such as c-fos). It is claimed that the more complex versions of these tasks model facets of ‘episodic-like’ memory; they may do, but they may reflect no more than different forms of associative familiarity with no re-experiencing of a past event.

In contrast, in a recall task, the animal must express behavior that reflects what it knows (non-episodic), or it remembers by re-experiencing a past event (episodic) in the absence of distinctive objects directly associated with what is being recalled. The former includes approaching one cryptic location rather than another in a spatial task. This is striking in the watermaze (Morris *et al*., 1982) because, from any cardinal starting position around the pool and using allocentric coding, a trained animal heads relatively directly to the correct stable position and lingers there searching for the platform until found, or if absent in a probe test, in a more extended but localised search. In effect, the animal heads directly for an object it cannot see, hear, or smell, and cannot feel until it has got there. The watermaze has, however, major limitations in that motivation cannot readily be manipulated nor is it suitable for electrophysiological, optical imaging or optogenetic procedures. In contrast, not only is the event arena much easier to use for these additional neuroscience techniques, but the level of deprivation of the animals and the reward incentive can be manipulated. In one study (Wang *et al*., 2010), a contrast is made between receiving a 1-pellet reward and a 3-pellets reward; in the former case there is clear forgetting over 24 h, whereas in the latter, memory of a specific daily location was above chance at 24 h.

A distinctive feature of the present protocols, which are based in part on the watermaze, is that the appearance of the apparatus at the start of any choice trial is identical – irrespective of whether the animal is in the stable target or recency target task. Extra-arena and two intra-arena cues stably define one context from the other across all sessions. The animal is in any of 3 start locations (E, S, or W) but, when the door opens to reveal the arena, the appearance of the arena and its intra-arena cues is unchanged apart from the ‘scene’ being rotationally different from each cardinal direction. The two tasks require carrying the reward from the correct sandwell to the north sidewall ‘homebase’; such carrying has been shown to be a natural behavior of rats (Whishaw *et al*., 1995) and the home-base definitively renders the task allocentric (Broadbent *et al*., 2020). What the animal must then do depends on the memory strategy being followed: it may recall the stable sandwell location appropriate to that context (Phase 1; ‘non-episodic’ memory); or it must go to the last location at which it found a rewarded sandwell in that context (Phase 2; a component of ‘episodic-like’ memory). Note that the startbox location used on a choice or probe trial is different from some of the starting locations of the sample trials. Thus, remembering an egocentrically directed path will not work.

There are other ingenious protocols for animals that examine recall. One example is a study by Eacott *et al*.. (2005) in which rats had to recall whether to turn left or right in a T-maze to approach a novel object that could *not* be seen (heard or smelled) at the choice point of either of two contexts. The animals could perform this ‘what-where-which’ task, and performance was disrupted by fornix lesions. A follow up study dissociated object familiarity from the ability to recall (Easton *et al*., 2009). Other similar examples include studies from Crystal Laboratory of ‘episodic-like’ memory in rodents based on studies of ‘foodstuff-location-time’ associations in the radial maze (Babb & Crystal, 2006; Crystal & Smith, 2014). Not only do these studies involve recall, but they also meet certain additional criteria that characterize a definitive animal model of episodic memory, including ‘what-where-which’ in the case of the Eacott studies. Our approach here shows definitively that the animals can successfully distinguish the two contexts (something that is implied but not shown directly in these other studies), that the animals can successfully recall the location in allocentric space where events have occurred in a specific context, and that the animals can do the episodic-like recency task repeatedly across sessions without interference. Our approach is, however, so far limited with respect to meeting ‘www’ criteria but follows the work of Howard Eichenbaum in using digging to secure reward (Fortin *et al*., 2004). This is more ‘event-like’ than merely inspecting an object, and the digging technique has been successfully shown to distinguish recollection and familiarity aspects of memory retrieval (Eichenbaum *et al*., 2007). We here meet a ‘context-location-event’ criterion.

Another issue concerns the use of a single-event versus multiple events. More recent work from the Crystal laboratory zeroes in elegantly addressing this issue and shows that multiple single events can be successfully encoded with respect to the context in which they occur (Panoz-Brown *et al*., 2016), and later recalled using a protocol that successfully distinguished between familiarity-based recognition of novelty from episodic-recall of item-in-context (Easton *et al*., 2009). The use of multiple single events as part of the daily “episode” in Phase 2 of our study (either 2 or 6 sample events) might be thought to undermine the status of our recency task in requiring episodic-like memory. We think this is unlikely for three reasons. First, certain tasks such as context fear conditioning are claimed to be “episodic” on the grounds that conditioning takes place in a single 3 min trial. In our view, this claim is a misunderstanding as Pavlovian conditioning can occur in a single trial and tests of this form of conditioning do not include any measures showing that the animals recall the conditioning experience; they merely freeze. Thus, having only a single trial is not a sufficient condition for episodic memory. Second, episodes often comprise multiple different events (e.g., remembering a children’s birthday party), but what makes the memory of such an event episodic is that the person has the experience of remembering it having happened before, in a particular context and consisting of a number of events (games, cake, candles, secret wishes etc.). Third, as emphasised by Crystal (2021), the encoding of an experience and the later recall of an episodic memory is not the expression of a learned behavior, but an experience of recalling something from the past. Our task meets this criterion because, on a choice trial in Phase 2, the animal is placed in either Context A or Context B and must first detect in which context it is located and then recall the cryptic spatial location where the event of digging for food has happened most recently. Such a situation might, in later experiments, allow for the possibility of an “unexpected test” (Zhou *et al*., 2012). The closest we got to this were our two unexpected NE choice tests in which the sample trials were not performed. Specifically, in our non-episodic recall task (Phase 1), the animal’s performance was excellent and unchanged, whereas performance fell to chance in the episodic task (Phase 2).

The use of a ‘recency’ protocol here might, nonetheless, be thought to include a cryptic familiarity component. One might argue that one area of space within each context becomes slightly more familiar during the 6 sample trials. In this case, upon exiting the start box, the animals in Phase 2 would not necessarily have to remember back to the prior events of those sample trials, but merely approach what feels like the most familiar part of the arena. This explanation is, however, unlikely for several reasons. First, the context of learning is completely familiar across more than 40 sessions of exposure, a situation that applies to both the extra-arena and intra-arena cues. Familiarity is usually studied using a novel object that is seen only once and this experience contrasted with a second trial in which the object is presented again together with a new ‘never-seen-before’ object. Rodents preferentially explore the novel object, becoming familiar with earlier novel objects in 1 or 2 exposures. Third, familiarity is a term that is usually applied to a specific stimulus, such as an object that can be approached; however, a location within a well learned, indeed, overtrained context representation cannot be considered a specific stimulus. There are no local cues and the correct sandwell is identical in visual appearance and olfactory cues to an incorrect one. The animals might, however, have adjusted the relative familiarity of a nearby local cue such as one of the two intra-arena cues. This is also unlikely as the intra-arena cues would have been seen as many as 92 times in each context (by the end of Phase 1). However, to test this, we measured and found no difference in the performance index when we compared trials in which local cues were adjacent to the correct sandwell (locations 3 and 4) to those in which they were more distant (locations 1, 2, 5 and 6; the apparatus was 1.6 m × 1.6 m; Fig. 3C-right). Moreover, familiarity is thought to decline with time, and our data shows (Fig. 3C-middle) that absolute familiarity did not mediate performance. We therefore argue that an episodic-like solution is the more reasonable interpretation: when the start door opens on a choice or probe trial, the animal must first judge his allocentric location at the edge of the context in front of him and then remember where he dug for food approximately 1.5–3 h earlier in that context. The human analogy would be to recognise that, in going from the kitchen to search for one’s glasses in the living room, it is not as if that location in the living room feels more familiar at the point of entering the room; one simply recalls an earlier event sitting on the sofa or at a table and searches for the glasses appropriately. Interestingly, the concept of “headed records” which we alluded to in the Introduction raises the interesting possibility that a record at the top of the pile may decay very little over time (Morton *et al*., 1985). If you haven’t seen a good friend for several years, and then meet up, you likely remember the place and circumstances of your last meeting however many days, weeks or years it was earlier. It would be interesting to test whether our context-specific spatial recall could successfully last a week, a month or longer.

The protocols described here are only a first step along the path to meeting the fuller ‘re-experiencing’ criteria of ‘episodic-like’ memory, with a distinctive feature so far being our ability to probe either non-episodic-like or episodic-like memory with no change of behavioral procedure on critical choice trials. Moreover, unlike spontaneous exploration studies of recognition memory, the control of inter-animal variability is arguably much better. In Phase 2 of this study, no less than Phase 1, every animal performed at an above chance level. There are several possible next steps. From a strictly behavioral perspective, it would as noted be valuable to know how long the stable and recency memory traces last. The prediction is that the latter might fade – but this time could still be quite long. A lesion study might reveal that both tasks are hippocampus-dependent, but an intriguing alternative would be if both required the hippocampus to be intact for *learning*, but only the recency task required an intact hippocampus for *recall*. There is the interesting possibility that so-called ‘hippocampal-dependent’ tasks are episodic-like in character during the initial stages of learning, but the temporal attributes become stripped from the memory traces as stability develops permitting systems consolidation and the mediation of recall by the neocortex without recollection. If the neocortex cannot ordinarily learn quickly, as the complementary learning systems idea supposes (McClelland *et al*., 1995), it is possible that the integrity of the hippocampus would not be required after consolidation unless the animal is seeking to update its memory or keep track of recency. A pharmacological study would open the opportunity to look at drugs that might affect memory encoding but not recall (e.g., intrahippocampal infusion of an NMDA receptor antagonist; (Morris, 1989)) and conversely, a drug that would limit recall even if only given just before the choice trial (e.g., an AMPA receptor antagonist (Bast *et al*., 2005)). Investigation of other brain areas would also be valuable (e.g., medial prefrontal cortex, retrosplenial cortex, and thalamus). For now, we seek to establish the viability of examining the context specificity of spatial memory in the domain of recall using two different protocols distinct from those used to study recognition memory.

## Materials and Methods

### Animals

Phase 1 of the study was conducted in two “replications” to provide an internal replication of stable context-specific spatial memory. There were 14 male Lister-hooded rats (Charles River, UK), aged 10–18 weeks and weighing 250–500 g (n = 8, cohort 1; n = 6, cohort 2). Phase 2 was conducted using the animals of cohort 2. The animals were maintained on a 12/12 h light-dark cycle, with behavioral training carried out in the light Phase at a controlled light intensity (110–120 lx) and temperature (20 ± 1 °C). During the training, the animals had *ad libitum* access to water, but access to food was restricted to maintain 85–90% of free-feeding body weight in reference to a normal growth curve. The study was conducted under a Project Licence to RM (P7AA53C3F) under the UK Animals (Scientific Procedures) Act 1986 and in compliance with University of Edinburgh regulations.

### Apparatus

#### Event arena

Training and behavioral tests were conducted in an “event arena” (an arena where events happen), consisting of a 160 cm^2^ open field with a floor comprised of a 7 × 7 grid of white removable tiles (20 cm^2^; Fig. 1B). Three black Plexiglas start-boxes (25 cm^3^), each with an automated door that was operable via an automated air pressure release system, were positioned centrally on three walls of the arena: located at East (E), South (S), and West (W; shaded orange and white in Fig1A). The goalbox located at N, which differed from the other start-boxes by having a closed ceiling (shaded blue), was used as a stable home-base to promote allocentric navigation (Broadbent *et al*., 2020).

The event arena could be configured as two distinct contexts (A and B), distinguishable by unique intra- and extra-arena cues (Fig. 1B). Context A consisted of a rabbit statue and lighthouse as the intra-arena cues; and 3D hanging objects, a grey blind, and a coloured photograph as extra-arena cues. Further, the arena walls (30 cm tall) were transparent in context A. Context B consisted of multi-coloured tennis balls and a fish statue as the intra-arena cues; occluding the extra-arena cues of context A, five grey blinds were drawn and used as the extra-arena cues in context B, as well as a blue cylindrical object in the N-W corner. Additionally, the grey blind drawn in context A was raised, leaving a white wall as an extra-arena cue in context B (Fig. 1B). There were black Plexiglas walls (30 cm tall) in context B.

#### Sandwells

Unflavored food rewards (0.5 g pellets; BioServ, UK) available as reward in the trials were buried in sandwells (described in detail in Broadbent *et al*., 2020) which could be inserted into one of six positions in the event arena. The sandwells had one accessible and one inaccessible compartment, with holes between the two allowing the passage of sand and odours. Irrespective of whether a sandwell was rewarded or non-rewarded during a trial, 16 pellets (16 in the inaccessible compartment, unrewarded; 12 in the inaccessible compartment and 4 in the accessible compartment, rewarded) were placed in the sandwells to radically reduce the likelihood of the animals using olfaction to guide performance. Sandwells were filled with sand and garam masala (4 kg: 6.5 g) to mask the scent of the pellets.

### Experimental protocol

The timeline over several weeks consisted of **habituation, Phase 1 training** (stable context-specific location task); **Phase 2 training** (context-specific spatial recency memory), and **control sessions**.

#### Habituation

After an acclimatisation period of 7 d, habituation sessions were conducted to familiarise the rats with the event arena, the presence of intra- and extra-arena cues, start-boxes, and specifically the sandwells in which they were taught to dig for food pellets. On habituation day 1 (HD1), the animals were given a food pellet in one of the start-boxes before exploring the arena for 5 min. On HDs 2–4, after receiving a pellet in a start-box, the rats were guided to locate a sandwell in which they dug for a pellet. After retrieving the pellet, the rats were guided to N to consume the food. On successive habituation days (HDs 5–8), the animals could retrieve two consecutive pellets from the sandwell, consuming both in N. The position of the sandwell changed daily and pellets were buried deeper to encourage digging behavior. All animals completed habituation successfully.

#### Phase 1: Context-specific location memory

Training sessions in the Phase 1 protocol began with 2 sample trials per context (as shown in Fig. 1A). A rewarded sandwell was placed at one of the 6 potential positions. Animals were placed in a start-box (E, S or W) with one 0.5 g pellet to eat as a cue signalling food availability in the arena. The door of the start-box was opened after 40 s allowing the rats to enter the arena. Once they had dug in the single available sandwell and retrieved a pellet (which Whishaw *et al*., (1995) have shown is spontaneously carried to a place of safety if above a certain size), the home-base door at north (N) was opened to permit entry with the reward pellet to be consumed there, the entrance door being closed behind the animal. After 30 s, the door of the home-base was re-opened, and the animals left to retrieve a second pellet from the same sample sandwell, confirming the encoding location, and again returned to N. This process of retrieving a pellet, entering N, re-entering the arena for a second pellet, and re-entering N was universal across the different types of trials. A 2^nd^ sample trial starting from a different start-box (e.g., E or W if the first was from S) was then conducted in the same context. After all animals had completed both sample trials in one context, the contextual configuration of the event arena was changed from A to B (or vice versa). The entire sequence of sample trials was then repeated in the other context. In this Phase 1 protocol, each animal was trained to retrieve food from only one location per context throughout their training (e.g., location 1 in context A and location 6 in context B). The animals were trained in groups such that all animals completed their sample trials in one context before being trained in the other, creating an interval of approximately 90 min between their first 2 sample trials in one context, and then their second 2 sample trials in the second context. Choice trials then commenced following the daily completion of the sample trials in sessions 2–11 and S13–22. During the daily choice trials, six sandwells (one rewarded and five non-rewarded) were placed in the arena (positions 1–6). The rewarded sandwell location corresponded with the sandwell location trained in the sample trials, specific to each context and counterbalanced with respect to locations across animals. The order of the contexts used during the choice trials was counterbalanced across sessions.

#### Performance measures

Each rat completed one choice trial per context. The time taken for the animal to dig in the correct sandwell (latency, s) and the number of errors were recorded for the retrieval of both pellets. In contrast, during probe trials (sessions 1, 12, and 23), six non-rewarded sandwells were placed in the arena. After consuming the cued pellet in the start-box, the animals were released from the start-box and the times spent digging in the correct and incorrect sandwell(s) were recorded during the first 120 s following the first dig (MultiTimer and LabView software). After 120 s, two pellets were placed in the correct sandwell for the animals to retrieve, helping to avoid any ‘extinction’ of the location of the correct sandwell. As in regular training, a session with probe trials included two sample trials in each context, followed by one probe trial per context.

#### Phase 2: Context-specific recency

The daily training protocol was identical to Phase 1 with two exceptions: (a) the location of the sandwell in the sample trial in each context changed every session, and thus the correct sandwell in the daily choice trial also changed each day; (b) following pilot work on the protocol, we switched from 3 sample trials/session/context to 6 sample trials/session/context (Fig. 1C). The animals’ task now was to find and remember the location of the sample trial sandwell and then recall its location during the daily probe test. Training sessions continued across a further 26 sessions. This protocol is procedurally every similar to Phase 1, but the task is episodic in character.

#### Control sessions

These sessions were non-encoding (NE) sessions in which the sample trials were not scheduled prior to the choice trials. They provided an internal check on our protocols. For Phase 1, the NE session served as a test of long-term context-specific location memory, as the rewarded sandwells were maintained at the same location across sessions. It was predicted that the animals would choose *correctly* during Phase 1. In contrast, the NE session for the recency protocol served as a test of whether the animals were inappropriately using another method other than recency memory to complete the task. One possibility would be the use of any olfactory cues from the rewarded sandwell, despite our efforts through sandwell design and masking to prevent this from happening. It was predicted that performance would be at *chance* for such a test during Phase 2.

## Highlights

- Rats can learn context-specific allocentric representations of spatial memory
- Rats can recall stable context-specific spatial locations of an event
- Rodents can also show context-specific recall of recent varying event locations
- Identical choice trials can distinguish between long-term and recency memory
